# Understanding and predicting synthetic lethal genetic interactions in *Saccharomyces cerevisiae *using domain genetic interactions

**DOI:** 10.1186/1752-0509-5-73

**Published:** 2011-05-17

**Authors:** Bo Li, Weiguo Cao, Jizhong Zhou, Feng Luo

**Affiliations:** 1 School of Computing, Clemson University, Clemson, SC 29634, USA; 2 Department of Genetics and Biochemistry, South Carolina Experiment Station, Clemson University, Clemson, SC 29634, USA; 3 Institue for Environmental Genomics, University of Oklahoma, Norman, OK, USA

## Abstract

**Background:**

Synthetic lethal genetic interactions among proteins have been widely used to define functional relationships between proteins and pathways. However, the molecular mechanism of synthetic lethal genetic interactions is still unclear.

**Results:**

In this study, we demonstrated that yeast synthetic lethal genetic interactions can be explained by the genetic interactions between domains of those proteins. The domain genetic interactions rarely overlap with the domain physical interactions from iPfam database and provide a complementary view about domain relationships. Moreover, we found that domains in multidomain yeast proteins contribute to their genetic interactions differently. The domain genetic interactions help more precisely define the function related to the synthetic lethal genetic interactions, and then help understand how domains contribute to different functionalities of multidomain proteins. Using the probabilities of domain genetic interactions, we were able to predict novel yeast synthetic lethal genetic interactions. Furthermore, we had also identified novel compensatory pathways from the predicted synthetic lethal genetic interactions.

**Conclusion:**

The identification of domain genetic interactions helps the understanding of originality of functional relationship in SLGIs at domain level. Our study significantly improved the understanding of yeast mulitdomain proteins, the synthetic lethal genetic interactions and the functional relationships between proteins and pathways.

## Background

Defining the functional relationships between proteins is one of the important tasks in the post-genomic era. A classical approach to understand gene functional relationships is producing combination mutant in two genes to observe genetic interactions [1]. Genetic interaction refers to the phenomenon in which the combined effect of mutations of two genes differs from individual effects of each mutation [2]. In the extreme cases, mutation of two nonessential genes could lead to a lethal phenotype. This kind of genetic interactions is referred as synthetic lethal genetic interactions (SLGIs). The genome-wide SLGIs have attracted much attention as they are capable of defining the genome-wide functional relationships between proteins, pathways and complexes [2-4]. The SLGIs also have potential for finding drug target or drug combinations [5].

Representing the structures and functions of proteins, protein domains are usually regarded as building blocks of proteins and are conserved during evolution. The mutation of a gene causes the loss of function of its protein product, which may accredit to the loss of protein domains in the protein product. Then, the effect of the mutation of two genes may be caused by the loss of protein domain combinations in both protein products. We refer the phenomenon in which combined effect of mutations of two domains in two proteins differs from individual effects of mutation of each domain as domain genetic interactions. The domain genetic interactions may correlate to SLGIs. We may use the domain genetic interactions to explain and predict the SLGIs. Furthermore, in multidomain proteins, different domains may fulfill different functions independently or collaboratively. Although genetic interaction analysis provides a promising method to understand the functional relationship between proteins [6], we cannot tell the contributions of different domains to certain functionality of multidomain proteins based on their genetic interactions. Studying the domain genetic interactions may help elucidate the SLGIs between multidomain proteins at domain level.

Genetic interactions are usually identified by mutant screens [7]. Recently, high throughput technologies, such as synthetic genetic arrays (SGA) [8] or synthetic lethal analysis by microarrays (SLAM) [9], have been developed for parallel and massive detection of genetic interactions. However, even with high throughput methods, experimental discovery of SLGIs is still overwhelming. Therefore, it is of interest to computationally predict SLGIs. Several computational approaches have been proposed for the prediction of SLGIs [10-15] and various features, such as protein interactions, gene expression, functional annotation, gene location, protein network characteristics, and genetic phenotype, had been utilized by these methods. Recently, Park et al. [15] also used protein domains as one of the features to predict interactions, including genetic interactions.

In our previous study [16], we performed a cross validation study on predicting SLGIs using protein domains as features. Our support vector machine (SVM) classifiers were able to achieve high performance with AUC (The area under the ROC curve) as 0.9272. These results suggested that using domain information may catch the genetic relationships between proteins. In this study, we first applied the Maximum Likelihood estimation (MLE) approach to estimate the probabilities of domain genetic interactions from yeast SLGIs. The EM algorithm developed by Dempster et al. [17] had been used to iteratively compute the maximum likelihood. Recently, the MLE method had been used to infer domain physical interactions from protein physical interactions [18,19]. We identified significant domain genetic interactions, which rarely overlap with the domain physical interactions from iPfam database [20]. We also showed that domains in multidomain yeast proteins contribute to their genetic interactions differently. Then, we were able to predict novel yeast SLGIs using probabilities of domain genetic interactions, which demonstrate the ability of protein domains in predicting SLGIs. We were also able to apply our predicted yeast SLGIs to understand the compensatory pathways. A complete description of our results and methods is given in the sections below.

## Results

### Identification of Significant Domain Genetic Interactions Using Maximum Likelihood Estimation

By assuming the independences among domain genetic interactions, we estimated the probabilities of domain genetic interactions using the Maximum Likelihood estimation (MLE) method. The probability of a domain pair indicates its propensity to genetically interact. The high probability of a domain pair imply that number of SLGIs including the domain interaction is high comparing to the number of protein pairs including the domain interaction that are not genetically interacted. Then, we calculated an evidence score E [19] for each domain genetic interaction to estimate its relative significance. The E scores are not the standard likelihood ratio test as they are calculated using only the positive data [19]. The E scores indicate the relative significances of the domain pairs in the SLGIs. The probability and the E score give different view about the significances of domain genetic interactions. The high probabilities do not always correlate to high E score. For example, there are a large number of protein pairs including a single domain pair. If very few of them are SLGIs, the probability of the domain pair will be low. However, if that domain pair is the only domain pair in those SLGIs, it will have high E score. On the other hand, in case there are a large number of protein pairs containing the same multiple domain pairs, if most of the protein pairs are SLGIs, the probabilities of those domain pairs will be high. However, the E scores of those domain pairs will be relatively low as removing any of those domain pairs will have limited effect on the probabilities of SLGIs. Therefore, we used both the probability and E score to select significant domain genetic interactions.

We first applied the MLE approach to the 7475 genetic interactions with both proteins having protein domains. We were able to assign probabilities and E scores to 11,189 domain pairs. Those domain genetic interactions are available on our website, http://www.genenetworks.net, for searching and downloading. We first selected the significant domain genetic interactions with E scores greater than or equal to 2.0, which corresponds to an approximate seven fold drop of the probability of SLGIs if this domain genetic interactions is excluded. Then, we selected the domain genetic interactions with probability greater than 0.5 even though they have low E score values. Totally, we obtained 3848 domain genetic interactions of 1027 domains. Table 1 lists top ten domain genetic interactions with the highest E scores. The probabilities of those ten domain genetic interactions vary from 0.013 to 0.8. The prefoldin domain dominates in the top ten domain genetic interactions (7 of 10). Our results are consistent with the findings of Le Meur and Gentleman [21], in which they showed that the prefoldin complex is in 9 of their top 10 pairs synthetic multi-protein complexes genetic interactions.

**Table 1:** Domain genetic interactions with top ten highest E scores.

E score	Prob.	Domain_a_	Domain_a _Pfam ID	# of Yeast proteins with domain_a_	Domain_b_	Domain_b _Pfam ID	# of Yeast proteins with domain_b_	# of GI
67.48	0.46	PF00225	Kinesin	6	PF01920	Prefoldin_2	4	11
65.59	0.72	PF01302	CAP_GLY	4	PF01920	Prefoldin_2	4	12
63.31	0.32	PF00022	Actin	9	PF01920	Prefoldin_2	4	11
41.73	0.025	PF00071	Ras	24	PF00071	Ras	24	13
39.53	0.036	PF00400	WD40	87	PF01920	Prefoldin_2	4	14
38.41	0.12	PF00071	Ras	24	PF04893	Yip1	3	8
36.53	0.027	PF00069	Pkinase	114	PF01920	Prefoldin_2	4	17
35.15	0.35	PF00225	Kinesin	6	PF02996	Prefoldin	3	6
33.16	0.80	PF01920	Prefoldin_2	4	PF03114	BAR	2	7
31.20	0.013	PF00022	Actin	9	PF00069	Pkinase	114	17

### Domain Genetic Interactions Rarely Overlap Domain Physical Interactions

To investigate the relationship between domain genetic interactions and the domain physical interactions, we compared the 3848 significant domain genetic interactions with the domain physical interactions from the iPfam database [20]. There are 4030 domain physical interactions of 1867 domains in iPfam database (2008 version). Among them, 1556 domain physical interactions of 1048 domains exist in yeast proteins we studied. There are 422 domains in both the genetic and physical interactions. We applied the Fisher's exact test in R to examine if domains participating genetic interactions are independent from domains participating in physical interactions. We obtained a p-value of 0.00025, which indicated a significant difference between two sets of protein domains. Furthermore, there are only 70 domain pairs overlapped between the 1556 domain physical interactions and 3848 domain genetic interactions. The Fisher's exact test obtained a p-value of 0, which showed that the domain genetic interactions significantly differ from the domain physical interactions. Thus, the domain genetic interaction is a new type of relationship among domains and can provide a complementary view about the relationships between domains.

### The Properties of Domain Genetic Interaction Network

**Figure 1:**
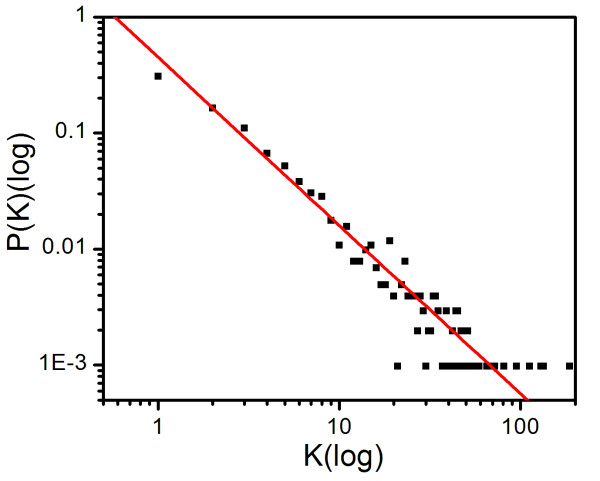
**The log-log plot of degree distribution P(K) of domain genetic interaction network**. The linear characteristics indicated by red line (y = x^-1.45^) imply that P(K) follows a power low.

To obtain an overview of the domain genetic interactions, we modeled the domain genetic interactions as a network, in which each node represents a domain and each link represents a genetic interaction between two domains. Then, we examined the properties of this domain genetic interaction network. The average connectivity of the network is 7.392. The domain PF00022 (Actin) has the highest connections of 186. The average shortest path among all nodes is 3.31. Comparing the size of nodes, this number is very small. Furthermore, the average node clustering coefficient of the network is 0.159. These properties indicated that the domain genetic interaction network has the small world property [22]. The analysis of the connectivity distribution of this network showed a power-law distribution with an exponent degree of 1.45 (Figure 1), which indicated that the domain genetic interaction network is a scale free network [23]. These results demonstrated that the domain genetic interaction network follows the common principles of biological networks [24].

### Domain Genetic Interactions in the SLGIs between Multidomain Proteins

Most proteins are multidomain proteins, which are created as a result of different genetic events, such as insertions and duplications [25,26]. Multidomain proteins may have different functionalities due to different domains. Our identification of domain genetic interactions helps understand how the domains contribute to functionality defined by the SLGIs, and then help elucidate the functional relationships between proteins at domain level from their genetic interactions.

**Figure 2:**
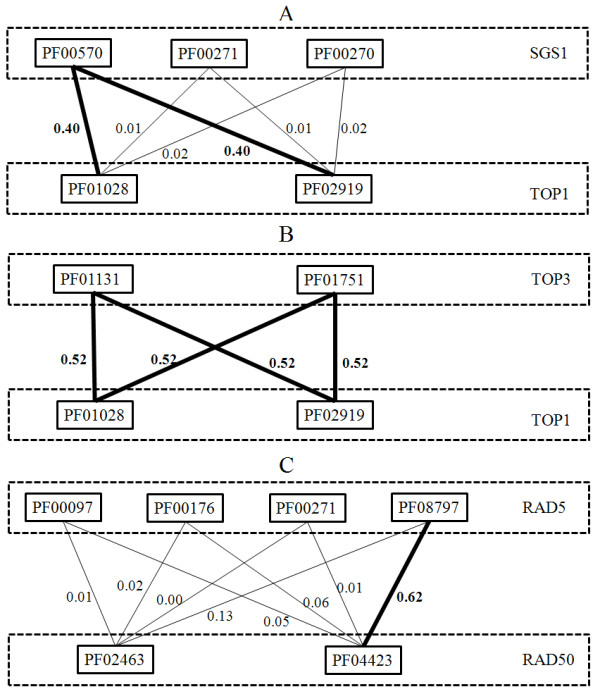
**The domain genetic interactions in SLGIs between multidomain proteins**. The thick lines indicate significant domain genetic interactions. The thin lines indicate the domain genetic interactions with low probability. The probabilities of domain genetic interactions are labeled besides the line.

Figure 2 shows examples of domain genetic interactions in three SLGIs. Figure 2A shows the domain genetic interactions between SGS1 and TOP1. The SGS1 has three domains and TOP1 has two domains. Only one of the three domains of SGS1, PF00570 (HRDC domain), has high probabilities to interact with two domains of TOP1: PF01028 (Eukaryotic DNA topoisomerase I catalytic core domain) and PF02919 (Eukaryotic DNA topoisomerase I DNA binding domain). The other two domains: PF00270 (DEAD/DEAH domain) and PF00271 (Helicase conserved C-terminal domain) of SGS1 have no genetic interaction with two domains of TOP1 (very low probabilities). Figure 2B shows the domain genetic interactions between TOP3 and TOP1. Both domains of TOP3: PF01131 (DNA topoisomerase domain) and PF01751 (Toprim domain) show high probabilities of genetic interaction with two domains of TOP1. Figure 2C shows the domain genetic interactions between RAD5 and RAD50. The RAD5 has four domains and RAD50 has two domains. The genetic interaction between RAD5 and RAD50 is mainly due to a single domain genetic interaction between PF08797 (HIRAN domain) of RAD5 and PF04423 (zinc hook domain) of RAD50. The PF00097 (Zinc finger domain), PF00176 (SNF2 family N-terminal domain) and PF00271 (Helicase conserved C-terminal domain) of RAD5 and PF02463 (RecF/RecN/SMC N terminal domain) of RAD50 have low contribution to the genetic interaction between RAD5 and RAD50. Those examples imply different domain genetic interaction architectures in SLGIs between yeast multidomaon proteins. The domain genetic interactions may exist between all domains of two proteins (TOP3 and TOP1), or between part of domains of one protein and all domains of the other protein (SGS1 and TOP1), or between part of domains of one protein and part of domains of the other protein (RAD5 and RAD50). The domain genetic interactions are able to help understand functional relationships between multidomain proteins at domain level.

We also investigated the domain genetic interactions of SLGIs between SGS1 and other proteins. We found that only the PF00570 (HRDC domain) of SGS1 has significant genetic interactions with other domains. The results implied that certain functionality of SGS1 may be only due to its HRDC domain, rather than its DEAD/DEAH domain and helicase conserved C-terminal domain. Previous study showed that the HRDC domain of SGS1 is required for its cellular functions involving topoisomerases [27]. Thus, the domain genetic interaction analysis can help understand how domains contribute to the different functionalities of multidomain proteins.

### Prediction and Validation of Genome-wide SLGIs Using Protein Domains

Having established that there is a strong correlation between domain genetic interactions and SLGIs, we explored to predict the probabilities of protein pairs to be SLGIs using probabilities of domain genetic interactions. We were able to assign 599752 protein pairs with probabilities greater than 0. Table S1 (Additional File 1) lists the number of SLGIs predicted by MLE approach at different probability cutoffs. All predicted SLGIs are hosted on our website for searching and downloading.

We then compared the correlation coefficients of gene expressions of predicted SLGIs to those of known SLGIs and those of all possible protein pairs. We used a yeast cell cycle gene expression data [28], which contains 77 data points. We calculated the T-score and P-value for the null hypothesis that there is no difference between the means of predicted SLGIs and the means of known SLGIs and the null hypothesis that there is no difference between the means of predicted SLGIs and the means of all pairs. The results are shown in Table S2 (Additional File 2). The correlation coefficients of gene expression of predicted SLGIs with threshold greater than 0.3 are similar to those of known SLGIs. The gene expression correlation coefficients of predicted SLGIs using different thresholds significantly differ from those of all pairs except for SLGIs with probabilities greater than 0.85, which have only small numbers of SLGIs. Those results indicated that the correlation coefficients of gene expressions of predicted SLGIs are similar to those of known SLGIs, rather than to those of random pairs. Recently, it was reported that the SLGIs are likely to have similar GO annotations [29]. We studied the distribution of similarities of Gene Ontology (GO) annotations between predicted SLGIs and also compared them to those of known SLGIs and all possible protein pairs. As shown in Table S3-5 (Additional File 3), the mean similarities of GO annotations of predicted SLGIs always significantly differ from those of all pairs. At certain probability thresholds, the mean similarities of GO annotations of predicted SLGIs show no significant differences from those of known SLGIs. Those thresholds are 0.35 for biological process and cellular component and 0.25 for molecular function. As the probability thresholds increase, the mean similarities of GO annotations also increase, which will make them differ from those of known SLGIs. The studies of GO annotations similarities and expression correlation coefficients showed that the predicted SLGIs at probability threshold around 0.3 are similar to experimentally obtained SLGIs.

### Novel SLGIs predicted by MLE Approach

**Figure 3:**
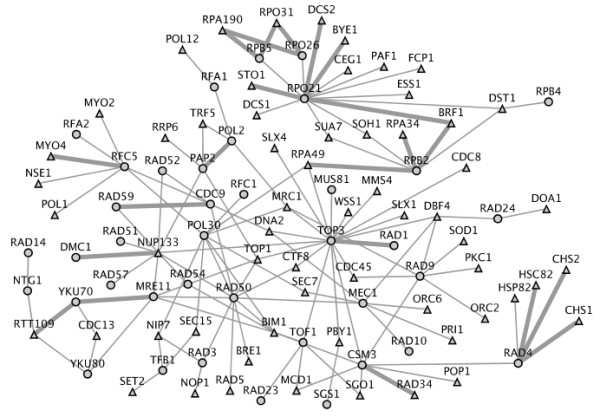
**The SLGIs (probabilities > 0.7) related to DNA repair proteins**. The cycles indicate DNA repair proteins and the triangle indicate non DNA repair proteins. Each genetic interaction involves at least one DNA repair protein. The wide links represent new genetic interactions. The figure was produced using Cytoscape [67].

The MLE approach was able to predict novel SLGIs. Table 2 lists 17 novel SLGIs (not included in our training data) with probability >0.9. We predicted the MYO4/DYN1 pair to be SLGI with the highest probability of 0.9895. The MYO4 is one of two type V myosin motors. The other one is MYO2, which is known to genetically interact with DYN1 [29]. Among 17 SLGIs, 12 SLGIs are between transcription initiation factor genes and genes from RNA polymerase complex. Previously, many SLGIs between transcription initiation factor genes and RNA polymerase genes have already been reported [30-32]. We expected our novel SLGIs to help further elucidate the transcription machine. We then investigated genes involved in cellular response to stresses caused by DNA damage. We downloaded a list of 116 DNA repair and recombination genes from Kyoto Encyclopedia of Genes and Genomes (KEGG) database [33]. Then, we extracted SLGIs in which at least one protein of SLGI pair are related to DNA repair. Figure 3 shows the DNA repair related SLGIs with probability > 0.7. Of a total of 133 SLGIs, 22 SLGIs are novel. Some of new predicted SLGIs are supported by previous studies. For example, the TOP3 and RAD1 double mutant has shown extreme synergistic growth defects in a previous study [34]. A recent study showed that the RTT109 and YKu70 double mutant exhibits synergistic defects under hydroxyurea treatment [35]. The PAP2 and POL2 were also shown to genetically interact at high temperatures [36].

**Table 2:** Predicted new synthetic lethal genetic interactions with probability greater than 0.9

Protein 1 Name	Protein 1 Annotation	Protein 2 Name	Protein 2 Annotation	Probability
MYO4	type V myosin motors	DYN1	Cytoplasmic heavy chain dynein	0.9895
RPO21	RNA polymerase II largest subunit	BRF1	TFIIIB B-related factor	0.9816
DST1	General transcription elongation factor TFIIS	RET1	Second-largest subunit of RNA polymerase III	0.9797
STO1	Large subunit of the nuclear mRNA cap-binding protein complex	RPA190	RNA polymerase I subunit	0.9586
STO1	Large subunit of the nuclear mRNA cap-binding protein complex	RPO21	RNA polymerase II largest subunit	0.9586
DST1	General transcription elongation factor TFIIS	RPA135	RNA polymerase I subunit A135	0.9570
BNR1	Formin, nucleates the formation of linear actin filaments	DYN1	Cytoplasmic heavy chain dynein	0.9441
CEG1	Alpha (guanylyltransferase) subunit of the mRNA capping enzyme	RPA190	RNA polymerase I subunit	0.9437
CEG1	Alpha (guanylyltransferase) subunit of the mRNA capping enzyme	RPO31	RNA polymerase III subunit	0.9437
RPB5	RNA polymerase subunit	RPA190	RNA polymerase I subunit	0.9437
RPB5	RNA polymerase subunit	RPO31	RNA polymerase III subunit	0.9437
DST1	General transcription elongation factor TFIIS	RPA190	RNA polymerase I subunit	0.9265
DST1	General transcription elongation factor TFIIS	RPO31	RNA polymerase III subunit	0.9265
ARO1	Pentafunctional arom protein	SPT16	Subunit of the heterodimeric FACT complex	0.9198
RET1	Second-largest subunit of RNA polymerase III	SUA7	Transcription factor TFIIB	0.9035
BRF1	TFIIIB B-related factor	RET1	Second-largest subunit of RNA polymerase III	0.9035
BRF1	TFIIIB B-related factor	RPB2	RNA polymerase II second largest subunit	0.9035

### Compensatory Pathways from Predicted SLGIs

Protein pathways are a part of gene network in the cell that can accomplish certain functionality. The SLGIs have been proposed to have high probability of occurrence in compensatory pathways [37]. Thus, the SLGIs within the pathways are rare and the SLGIs between pathways are significantly abundant. Identification compensatory pathways from synthetic lethal genetic interactions can be a powerful way to understanding cellular functional relationships. We expected our new predictions will increase the ability of understanding compensatory pathways. We applied the algorithm of Ma [38] to identify compensatory pathways from 7583 predicted SLGIs with probability higher than 0.3. Among 7583 SLGIs, 4497 SLGIs are novel predictions. Although Ma et al. have shown that physical interactions are enriched in discovered pathways [38], there is no assumption that proteins in those pathways are physically interacting. Totally, we obtained 167 pairs of compensatory pathways, which include 638 proteins and 3535 SLGIs. Then, we examined the GO term co-occurrences in each pathway using the SGD GO Term Finder [39]. The GO Term Finder calculated the p-values that reflect the probability of observing the co-occurrence of proteins with a given GO term in a certain pathway by chance based on a binomial distribution. Among 167 compensatory pathway pairs, 153 pairs are found significantly enriched GO terms with p-value less than 0.05. All 167 pairs of compensatory pathways and their most significant GO terms are listed on our http://www.genenetworks.net website.

**Figure 4:**
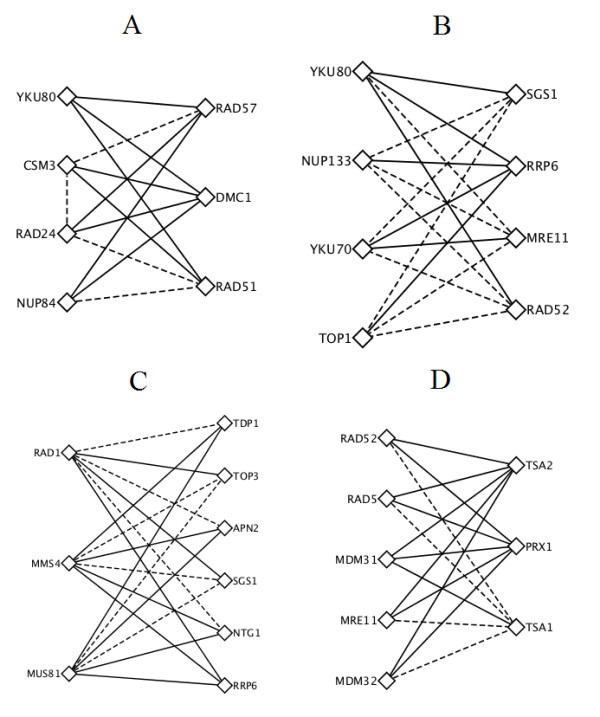
**Compensatory pathways identified from predicted SLGIs**. (A, B) two compensatory pathways related to DNA double strand breaks repairs. Pathways on the left of A and B belong to non-homologous end joining. Pathways on the right of A and B belong to homologous recombination. (C) A compensatory pathways related to DNA repairs. (D) A compensatory pathways related to hydroperoxides response in the cell. Dashed lines indicate known SLGIs and solid lines indicated novel predicted SLGIs. The figure was produced using Cytoscape [67].

Figure 4A and 4B list two pairs of compensatory pathways related to DNA double strand break (DSB) repair. Among total 28 SLGIs between those two compensatory pathways, only 12 of them are known. The DSB is a kind of lethal DNA damage in which both strands of double helix are cleaved. The cell maintains multiple mechanisms to repair double strand breaks with the homologous recombination (HR) and non-homologous end joining (NHEJ) as two major mechanisms. Pathways involving those two mechanisms had shown to be compensatory to each other in Drosophila [40]. The pathways on the left of two compensatory pairs involve in NHEJ. The KU complex (YKU80 and YKU70) is the damage detector of NHEJ [41]. Mutation of nucleoporins NUP84 and NUP133 was reported to be hypersensitive to DNA damage [42]. Furthermore, the nucleoporins (NUP84 and NUP133) had been reported to colocalize and coimmunoprecipitate with Slx5/Slx8 [43], which regulate a DNA repair pathway that counteract Rad5-independent HR [44]. Our discovery suggested that the nucleoporins (NUP84 and NUP133) may regulate the NHEJ pathway through Ku complex for double strand breaks repair. The RAD24 is a DNA damage checkpoint protein [45]. Studies also had shown that the YKU80 and RAD24 are in the same NHEJ pathways [45] to repair irradiation and methylmethanesulphonate (MMS) damages. The CSM3 is a DNA replication checkpoint protein. Genetic interaction between RAD24 and CSM3 may indicate the pathway on the right side of Figure 4 A can actually become two pathways: one is YKU80, RAD24, NUP84; the other is YKU80, CSM3 and NUP84.

The pathways on the right of those two compensatory pairs of Figure 4 A and 4B involve in HR. On the right of Figure 4A, the pathway involves three proteins: RAD57, RAD51 and DMC1. The DMC1 and RAD51 are known to form a complex [46] and have roles in recombination [47]. RAD51 and RAD57 are in the same protein family and it has been shown that RAD57/RAD55 bind with RAD51 [48]. On the right of Figure 4B, the pathway involves four proteins: SGS1, RRP6, MRE11 and RAD52. The MRE11 and SGS1 are part of a two-step mechanism to initial HR [49]. The RAD52 plays a major role in the single strand annealing and strand exchange.

Figure 4C also shows two compensatory DNA repair pathways. Among 18 SLGIs between two pathways, 11 are novel predictions. Many previous studies have supported our prediction of compensatory functionalities of those two pathways. Guillet and Boiteus [50] reported that the APN2 and MUS81/MMS4 have overlapping function to repair 3'-blocked single strand breaks (SSBs). Vance and Wilson [51] showed that TDP1and RAD1 function as redundant pathways. SGS1/TOP3 had also been reported to have overlapping functionality with the MMS4/MUS81 [52]. Proteins in these compensatory pathways involve in many DNA-repair pathways, such as base excision repair (BER), nucleotide excision repair (NER) and HR. Studies already showed that BER, NER and HR pathways have overlapping specificities [53]. Our prediction of these compensatory pathways may help to understand the overlapping functionalities among BER, NER and HR pathways.

Another interesting compensatory pathway pair is related to hydroperoxides response in the cell. As shown in Figure 4D, 11 of 15 SLGIs connecting two pathways are novel predictions. The three proteins (TSA1, TSA2 and PRX1) on the right are all thioredoxin peroxidase. They play a role in reduction of hydroperocxides in cytoplasm (TSA1 and TSA2) and mitochondrion (PRX1). Meanwhile, the five proteins (RAD52, RAD5, MDM31, MDM32, and MRE11) in the left pathway are related to DNA repair. RAD52, RAD5 and MRE11 are known related to double strand break repair [54-56]. The MDM31 and MDM32 relate the stability of mitochondrial DNA [57]. Those five proteins may relate to repair the damage created by the hydroperoxides. This compensatory pathway pair implies that removing hydroperoxides or repairing damage is alternative strategies for cells to survive from hydropeoxides.

## Discussion and Conclusions

In this study, we demonstrated that the synthetic lethal genetic interactions between two proteins can be described by the genetic interactions between domains of those proteins. We applied a probabilistic model to successfully identify significant domain genetic interactions. The significant domain genetic interactions rarely overlap with the domain physical interactions from iPfam [20], which suggests that the domain genetic interactions and domain physical interactions are complementary to each other. The domain genetic interactions offer a new understanding of the relationship among domains, and then among proteins. Furthermore, with the identified domain genetic interactions, we showed that the contributions of domains in a multidomain protein to its genetic interactions are significantly different. The domain genetic interactions will help to decipher the domains that perform the function related to the genetic interaction. For example, analyzing the domain genetic interactions confirms that only the PF00570 (HRDC domain) of SGS1 involve in the cellular functions that are compensatory to topoisomerases.

Our identification of domain genetic interactions and prediction of SLGIs is not complete. First, our training data is limited. It is believed that the available genetic interactions are just a small fraction of the whole genetic interactions [58]. Second, the training data largely come from several high-throughput genetic interaction screens, which emphasized certain biological processes, such as DNA repair. Thus, the probability of domain genetic interaction may be effected by the future available SLGIs. Moreover, we should keep in mind that our predicted SLGIs require further validation to exclude false positives. The MLE approach assumes the independence of domain genetic interactions. However, there may be dependence between domain genetic interactions. An apparent extension of this work is to consider the dependences among cooperative domains in multidomain proteins. Due to those limitations, the performance of predicting genome-wide SLGIs using protein domains may be not as good as other classifiers [11].

In summary, our study significantly improved the understanding of different domains in mulitdomain proteins. The identification of domain genetic interactions helps the understanding of originality of functional relationship in SLGIs at domain level. Furthermore, our prediction of SLGIs expanded the ability to elucidate the functional relationships between proteins and pathways.

## Methods

### Source of Data

We collected the protein domain data from Pfam (Protein families database) [59]. The Pfam database provides two types of protein family data. Pfam-A domains are manually curated while Pfam-B domains are automatically generated. In our study, only Pfam-A domains were considered. The total number of selected Pfam-A domains for yeast is 2289. We downloaded the genetic interactions of yeast from the Saccharomyces Genome Database (SGD) [39] (July 2009 version), which were compiled from different biological experiments [8,60-66]. Then, we extracted synthetic lethal interaction data set from the file containing all the genetic interactions. After removing replicates, there were totally 10977 pairs of synthetic lethal interactions of 2640 proteins. We removed protein pairs from study if either protein in the pair does not contain any domain. Eventually, we obtained 7475 synthetic lethal interactions among 2008 proteins. There were 1357 unique domains in those 7475 SLGIs. The information of SLGIs used in this study is summarized in Table 3.

**Table 3:** Summary of SLGIs used in this study

Number of known SLGIs with no replicates	10977
Number of unique proteins in known SLGIs	2640
Number of SLGIs between proteins with domains	7475
Number of unique proteins in selected SLGIs	2008
Number of unique domains in selected SLGIs	1357

### Estimation of probabilities and significances of domain genetic interactions

We treated the protein genetic interactions *P*_*m,n *_and domain genetic interaction *D*_*i,j *_as random variables. The *P*_*m,n *_= 1 if two proteins *i *and *j *genetically interact and *P*_*m,n *_= 0 otherwise. The *D*_*i,j *_= 1 if two domain *i *and *j *genetically interact and *D*_*i,j *_= 0 otherwise. We estimated the probabilities of potential domain interactions Pr(*D*_*i,j *_= 1) by maximizing the likelihood of observed genetic interactions using the Expectation-Maximization (EM) algorithm [17-19]. The EM algorithm iteratively estimates the maximum likelihood of the 'complete data' that combine the observed data and unobserved data. Here, the protein genetic interactions and the domain information of proteins are our observed data and the domain genetic interactions are our unobserved data. By assuming the independences among domain genetic interactions, the likelihood of observed protein genetic interactions based on domain genetic interactions can be obtained as:(1)

where M_i,j _be the number of genetic interacting pairs between domain i and j in all protein genetic interactions; N_i,j _be the number of non genetic interacting domain pairs between domain i and j in protein genetic interactions; and K_i,j _be the number of non genetic interacting protein pairs including domain i in one protein and j in the other one. The value of K_i,j _is counted from all possible protein pairs with domain i in one protein and j in the other one with excluding the known genetic interacting protein pairs. The K_i,j _will keep unchanged during EM computation. The *a *and *b *are pseudo counts to avoid the Pr(D_i,j _= 1) or Pr(D_i,j _= 0) to be zero when instances of domains i and j are rare. We set both *a *and *b *to 1 in our calculation. Initially, the M_i,j _was set to the number of genetic interactions between domain i and j in experimental genetic interactions; N_i,j _is set to 0. And Pr(D_i,j _= 1) was initialized as following:(2)

In each Expectation step of EM algorithm, we first estimated the expected values of E[M_i,j_] and E[N_i,j_] [19] using the current Pr(D_i,j _= 1):(3)(4)

Then, we calculate the Pr(D_i,j _= 1) using the E[M_i,j_] and E[N_i,j_] as following (Maximization step):(5)

The EM algorithm was iterated the above Expectation and Maximization steps till the change of likelihood L is less than a pre-defined small value.

The evidence score E_i,j _[19] of domain pair i and j is defined as the ratio between the probability that a pair of proteins, m and n, genetically interact given that the pair of domains, i and j, genetically interact and the probability that a pair of proteins, m and n, genetically interact given that the pair of domains, i and j, do not genetically interact:(6)

The  denotes the probability of genetic interacting between domains k and l given that the domains i and j do not genetically interact.

Prediction of SLGIs using probabilities of domain genetic interactions

We assumed that two proteins genetically interact (*P*_*m,n *_= 1) if and only if at least one domain pair from the two proteins genetically interact (*D*_*i,j *_= 1). Then, we calculated the probability of two proteins genetically interacting Pr(*P*_*m,n *_= 1) as following:(7)

A pair of proteins was predicted to be SLGI only if its probability is higher than a predefined threshold.

## Authors' contributions

FL designed research. BL implemented the idea. FL, BL, WGC and JZH analyzed the results. All authors have read and approved the final manuscript.

## Supplementary Material

Additional file 1 **Table S1**. Number of predicted SLGIs by MLE approach using different posterior probability threshold Click here for file

Additional file 2 **Table S2**. Summary statistics of distribution of the correlation coefficient between the cell cycle expression profiles of genetic interacting proteins Click here for file

Additional file 3 **Table S3-5**. Summary statistics of distribution of the similarities between the GO biological processes, cellular component and molecular function of genetic interacting proteins Click here for file

## References

[B1] Hartman J Garvik B Hartwell L Principles for the buffering of genetic variation Science 2001 291 1001 1004 10.1126/science.291.5506.1001 11232561

[B2] Mani R St Onge RP Hartman JL Giaever G Roth FP Defining genetic interaction Proceedings of the National Academy of Sciences 2008 105 9 3461 3461 10.1073/pnas.0712255105 PMC2265146 18305163

[B3] Ye P Peyser BD Pan X Boeke JD Spencer FA Bader JS Gene function prediction from congruent synthetic lethal interactions in yeast Molecular Systems Biology 2005 1 1 10.1038/msb4100034 PMC1681444 16729061

[B4] Wong SL Zhang LV Roth FP Discovering functional relationships: biochemistry versus genetics Trends Genet 2005 21 8 424 427 10.1016/j.tig.2005.06.006 15982781

[B5] Kaelin WG Jr The concept of synthetic lethality in the context of anticancer therapy Nat Rev Cancer 2005 5 9 689 698 10.1038/nrc1691 16110319

[B6] Ooi SL Pan X Peyser BD Ye P Meluh PB Yuan DS Irizarry RA Bader JS Spencer FA Boeke JD Global synthetic-lethality analysis and yeast functional profiling Trends Genet 2006 22 1 56 63 10.1016/j.tig.2005.11.003 16309778

[B7] Winzeler EA Shoemaker DD Astromoff A Liang H Anderson K Andre B Bangham R Benito R Boeke JD Bussey H Functional characterization of the S. cerevisiae genome by gene deletion and parallel analysis Science 1999 285 5429 901 906 10.1126/science.285.5429.901 10436161

[B8] Tong AH Evangelista M Parsons AB Xu H Bader GD Page N Robinson M Raghibizadeh S Hogue CW Bussey H Systematic genetic analysis with ordered arrays of yeast deletion mutants Science 2001 294 5550 2364 2368 10.1126/science.1065810 11743205

[B9] Pan X Yuan DS Ooi SL Wang X Sookhai-Mahadeo S Meluh P Boeke JD dSLAM analysis of genome-wide genetic interactions in Saccharomyces cerevisiae Methods 2007 41 2 206 221 10.1016/j.ymeth.2006.07.033 17189863 PMC2491713

[B10] Chipman KC Singh AK Predicting genetic interactions with random walks on biological networks BMC Bioinformatics 2009 10 17 10.1186/1471-2105-10-17 19138426 PMC2653491

[B11] Qi Y Suhail Y Lin YY Boeke JD Bader JS Finding friends and enemies in an enemies-only network: a graph diffusion kernel for predicting novel genetic interactions and co-complex membership from yeast genetic interactions Genome Res 2008 18 12 1991 2004 10.1101/gr.077693.108 18832443 PMC2593582

[B12] Wong SL Zhang LV Tong AHY Li Z Goldberg DS King OD Lesage G Vidal M Andrews B Bussey H Combining biological networks to predict genetic interactions Proceedings of the National Academy of Sciences 2004 101 44 15682 15687 10.1073/pnas.0406614101 PMC524818 15496468

[B13] Paladugu S Zhao S Ray A Raval A Mining protein networks for synthetic genetic interactions BMC Bioinformatics 2008 9 1 426 426 10.1186/1471-2105-9-426 18844977 PMC2577120

[B14] Zhong W Sternberg PW Genome-wide prediction of C. elegans genetic interactions Science 2006 311 5766 1481 1484 10.1126/science.1123287 16527984

[B15] Park CY Hess DC Huttenhower C Troyanskaya OG Simultaneous genome-wide inference of physical, genetic, regulatory, and functional pathway components PLoS Comput Biol 2010 6 11 e1001009 10.1371/journal.pcbi.1001009 21124865 PMC2991250

[B16] Li B Luo F Predicting yeast synthetic lethal genetic interactions using protein domains IEEE International Conference on Bioinformatics and Biomedicine (BIBM). Washington DC 2009 43 47

[B17] Dempster AP Laird NM Rubin DB Maximum likelihood from incomplete data via the EM algorithm Journal of the Royal Statistical Society Series B (Methodological) 1977 39 1 1 38

[B18] Deng M Mehta S Sun F Chen T Inferring domain-domain interactions from protein-protein interactions Proceedings of the sixth annual international conference on Computational biology: 2002; Washington, DC, USA 2002 ACM 117 126

[B19] Riley R Lee C Sabatti C Eisenberg D Inferring protein domain interactions from databases of interacting proteins Genome Biology 2005 6 10 R89 R89 10.1186/gb-2005-6-10-r89 16207360 PMC1257472

[B20] Finn RD Marshall M Bateman A iPfam: visualization of protein-protein interactions in PDB at domain and amino acid resolutions Bioinformatics 2005 21 3 410 412 10.1093/bioinformatics/bti011 15353450

[B21] Le Meur N Gentleman R Modeling synthetic lethality Genome Biology 2008 9 9 R135 10.1186/gb-2008-9-9-r135 18789146 PMC2592713

[B22] Watts DJ Strogatz SH Collective dynamics of 'small-world' networks Nature 1998 393 6684 440 442 10.1038/30918 9623998

[B23] Barabási AL Albert R Emergence of Scaling in Random Networks Science 1999 286 5439 509 512 10.1126/science.286.5439.509 10521342

[B24] Barabasi AL Oltvai ZN Network biology: understanding the cell's functional organization Nat Rev Genet 2004 5 2 101 113 10.1038/nrg1272 14735121

[B25] Apic G Gough J Teichmann SA Domain combinations in archaeal, eubacterial and eukaryotic proteomes J Mol Biol 2001 310 2 311 325 10.1006/jmbi.2001.4776 11428892

[B26] Vogel C Teichmann SA Pereira-Leal J The relationship between domain duplication and recombination J Mol Biol 2005 346 1 355 365 10.1016/j.jmb.2004.11.050 15663950

[B27] Mullen JR Kaliraman V Brill SJ Bipartite structure of the SGS1 DNA helicase in Saccharomyces cerevisiae Genetics 2000 154 3 1101 1114 10757756 10.1093/genetics/154.3.1101 PMC1460973

[B28] Spellman PT Sherlock G Zhang MQ Iyer VR Anders K Eisen MB Brown PO Botstein D Futcher B Comprehensive identification of cell cycle-regulated genes of the yeast Saccharomyces cerevisiae by microarray hybridization Mol Biol Cell 1998 9 12 3273 3297 9843569 10.1091/mbc.9.12.3273 PMC25624

[B29] Tong AHY Lesage G Bader GD Ding H Xu H Xin X Young J Berriz GF Brost RL Chang M Global mapping of the yeast genetic interaction network Science 2004 303 5659 808-813-808-813 10.1126/science.1091317 14764870

[B30] Wilcox CB Rossettini A Hanes SD Genetic Interactions With C-Terminal Domain (CTD) Kinases and the CTD of RNA Pol II Suggest a Role for ESS1 in Transcription Initiation and Elongation in Saccharomyces cerevisiae Genetics 2004 167 1 93 105 10.1534/genetics.167.1.93 15166139 PMC1470855

[B31] Malagon F Tong AH Shafer BK Strathern JN Genetic interactions of DST1 in Saccharomyces cerevisiae suggest a role of TFIIS in the initiation-elongation transition Genetics 2004 166 3 1215 1227 10.1534/genetics.166.3.1215 15082542 PMC1470799

[B32] Archambault J Lacroute F Ruet A Friesen JD Genetic interaction between transcription elongation factor TFIIS and RNA polymerase II Mol Cell Biol 1992 12 9 4142 4152 1508210 10.1128/mcb.12.9.4142-4152.1992 PMC360315

[B33] Kanehisa M Goto S Kawashima S Okuno Y Hattori M The KEGG resource for deciphering the genome Nucleic Acids Res 2004 32 Database D277 280 10.1093/nar/gkh063 PMC308797 14681412

[B34] Shor E Gangloff S Wagner M Weinstein J Price G Rothstein R Mutations in Homologous Recombination Genes Rescue top3 Slow Growth in Saccharomyces cerevisiae Genetics 2002 162 2 647 662 12399378 10.1093/genetics/162.2.647 PMC1462310

[B35] Jessulat M Alamgir M Salsali H Greenblatt J Xu J Golshani A Interacting proteins Rtt109 and Vps75 affect the efficiency of non-homologous end-joining in Saccharomyces cerevisiae Arch Biochem Biophys 2008 469 2 157 164 10.1016/j.abb.2007.11.001 18036332

[B36] Edwards S Li CM Levy DL Brown J Snow PM Campbell JL Saccharomyces cerevisiae DNA Polymerase {varepsilon} and Polymerase {sigma} Interact Physically and Functionally, Suggesting a Role for Polymerase {varepsilon} in Sister Chromatid Cohesion Mol Cell Biol 2003 23 8 2733 2748 10.1128/MCB.23.8.2733-2748.2003 12665575 PMC152548

[B37] Kelley R Ideker T Systematic interpretation of genetic interactions using protein networks Nature biotechnology 2005 23 5 561 566 10.1038/nbt1096 15877074 PMC2814446

[B38] Ma X Tarone AM Li W Mapping Genetically Compensatory Pathways from Synthetic Lethal Interactions in Yeast PLoS ONE 2008 3 4 e1922 10.1371/journal.pone.0001922 18398455 PMC2275788

[B39] Cherry JM Adler C Ball C Chervitz SA Dwight SS Hester ET Jia Y Juvik G Roe T Schroeder M SGD: Saccharomyces genome database Nucleic Acids Research 1998 26 1 73 73 10.1093/nar/26.1.73 9399804 PMC147204

[B40] Johnson-Schlitz DM Flores C Engels WR Multiple-Pathway Analysis of Double-Strand Break Repair Mutations in Drosophila PLoS Genet 2007 3 4 e50 10.1371/journal.pgen.0030050 17432935 PMC1851981

[B41] Pastwa E Blasiak J Non-homologous DNA end joining Acta Biochim Pol 2003 50 891 908 14739985

[B42] Therizols P Fairhead Cc Cabal GG Genovesio A Olivo-Marin JC Dujon B Fabre E Telomere tethering at the nuclear periphery is essential for efficient DNA double strand break repair in subtelomeric region The Journal of Cell Biology 2006 172 2 189 199 10.1083/jcb.200505159 16418532 PMC2063549

[B43] Nagai S Dubrana K Tsai-Pflugfelder M Davidson MB Roberts TM Brown GW Varela E Hediger F Gasser SM Krogan NJ Functional Targeting of DNA Damage to a Nuclear Pore-Associated SUMO-Dependent Ubiquitin Ligase Science 2008 322 5901 597 602 10.1126/science.1162790 18948542 PMC3518492

[B44] Burgess RC Rahman S Lisby M Rothstein R Zhao X The Slx5-Slx8 Complex Affects Sumoylation of DNA Repair Proteins and Negatively Regulates Recombination Mol Cell Biol 2007 27 17 6153 6162 10.1128/MCB.00787-07 17591698 PMC1952148

[B45] de la Torre-Ruiz M-A Lowndes NF The Saccharomyces cerevisiae DNA damage checkpoint is required for efficient repair of double strand breaks by non-homologous end joining FEBS Letters 2000 467 2-3 311 315 10.1016/S0014-5793(00)01180-7 10675560

[B46] Bishop DK RecA homologs Dmc1 and Rad51 interact to form multiple nuclear complexes prior to meiotic chromosome synapsis Cell 1994 79 6 1081 1092 10.1016/0092-8674(94)90038-8 7528104

[B47] Masson JY West SC The Rad51 and Dmc1 recombinases: a non-identical twin relationship Trends in Biochemical Sciences 2001 26 2 131 136 10.1016/S0968-0004(00)01742-4 11166572

[B48] Sung P Yeast Rad55 and Rad57 proteins form a heterodimer that functions with replication protein A to promote DNA strand exchange by Rad51 recombinase Genes & Development 1997 11 9 1111 1121 10.1101/gad.11.9.1111 9159392

[B49] Mimitou EP Symington LS Sae2, Exo1 and Sgs1 collaborate in DNA double-strand break processing Nature 2008 455 7214 770 774 10.1038/nature07312 18806779 PMC3818707

[B50] Guillet M Boiteux S Endogenous DNA abasic sites cause cell death in the absence of Apn1, Apn2 and Rad1/Rad10 in Saccharomyces cerevisiae EMBO J 2002 21 11 2833 2841 10.1093/emboj/21.11.2833 12032096 PMC126023

[B51] Vance JR Wilson TE Yeast Tdp1 and Rad1-Rad10 function as redundant pathways for repairing Top1 replicative damage Proceedings of the National Academy of Sciences of the United States of America 2002 99 21 13669 13674 10.1073/pnas.202242599 12368472 PMC129737

[B52] Kaliraman V Mullen JR Fricke WM Bastin-Shanower SA Brill SJ Functional overlap between Sgs1-Top3 and the Mms4-Mus81 endonuclease Genes & Development 2001 15 20 2730 2740 10.1101/gad.932201 11641278 PMC312806

[B53] Swanson RL Morey NJ Doetsch PW Jinks-Robertson S Overlapping Specificities of Base Excision Repair, Nucleotide Excision Repair, Recombination, and Translesion Synthesis Pathways for DNA Base Damage in Saccharomyces cerevisiae Mol Cell Biol 1999 19 4 2929 2935 10082560 10.1128/mcb.19.4.2929 PMC84087

[B54] D'Amours D Jackson SP The Mre11 complex: at the crossroads of dna repair and checkpoint signalling Nat Rev Mol Cell Biol 2002 3 5 317 327 10.1038/nrm805 11988766

[B55] Lisby M Rothstein R Mortensen UH Rad52 forms DNA repair and recombination centers during S phase Proc Natl Acad Sci USA 2001 98 15 8276 8282 10.1073/pnas.121006298 11459964 PMC37432

[B56] Johnson RE Prakash S Prakash L Yeast DNA repair protein RAD5 that promotes instability of simple repetitive sequences is a DNA-dependent ATPase J Biol Chem 1994 269 45 28259 28262 7961763

[B57] Dimmer KS Jakobs S Vogel F Altmann K Westermann B Mdm31 and Mdm32 are inner membrane proteins required for maintenance of mitochondrial shape and stability of mitochondrial DNA nucleoids in yeast J Cell Biol 2005 168 1 103 115 15631992 10.1083/jcb.200410030 PMC2171677

[B58] Boone C Bussey H Andrews BJ Exploring genetic interactions and networks with yeast Nature Reviews Genetics 2007 8 6 437 449 10.1038/nrg2085 17510664

[B59] Bateman A Birney E Cerruti L Durbin R Etwiller L Eddy SR Griffiths-Jones S Howe KL Marshall M Sonnhammer ELL The Pfam protein families database Nucleic acids research 2002 30 1 276 276 10.1093/nar/30.1.276 11752314 PMC99071

[B60] Fortes P Kufel J Fornerod M Polycarpou-Schwarz M Lafontaine D Tollervey D Mattaj IW Genetic and physical interactions involving the yeast nuclear cap-binding complex Mol Cell Biol 1999 19 10 6543 6553 10490594 10.1128/mcb.19.10.6543 PMC84624

[B61] Tong AH Lesage G Bader GD Ding H Xu H Xin X Young J Berriz GF Brost RL Chang M Global mapping of the yeast genetic interaction network Science 2004 303 5659 808 813 10.1126/science.1091317 14764870

[B62] Krogan NJ Kim M Tong A Golshani A Cagney G Canadien V Richards DP Beattie BK Emili A Boone C Methylation of histone H3 by Set2 in Saccharomyces cerevisiae is linked to transcriptional elongation by RNA polymerase II Mol Cell Biol 2003 23 12 4207 4218 10.1128/MCB.23.12.4207-4218.2003 12773564 PMC427527

[B63] Huang D Moffat J Andrews B Dissection of a complex phenotype by functional genomics reveals roles for the yeast cyclin-dependent protein kinase Pho85 in stress adaptation and cell integrity Mol Cell Biol 2002 22 14 5076 5088 10.1128/MCB.22.14.5076-5088.2002 12077337 PMC139770

[B64] Goehring AS Mitchell DA Tong AH Keniry ME Boone C Sprague GF Jr Synthetic lethal analysis implicates Ste20p, a p21-activated potein kinase, in polarisome activation Mol Biol Cell 2003 14 4 1501 1516 10.1091/mbc.E02-06-0348 12686605 PMC153118

[B65] Kozminski KG Beven L Angerman E Tong AH Boone C Park HO Interaction between a Ras and a Rho GTPase couples selection of a growth site to the development of cell polarity in yeast Mol Biol Cell 2003 14 12 4958 4970 10.1091/mbc.E03-06-0426 12960420 PMC284798

[B66] Davierwala AP Haynes J Li Z Brost RL Robinson MD Yu L Mnaimneh S Ding H Zhu H Chen Y The synthetic genetic interaction spectrum of essential genes Nat Genet 2005 37 10 1147 1152 10.1038/ng1640 16155567

[B67] Shannon P Markiel A Ozier O Baliga NS Wang JT Ramage D Amin N Schwikowski B Ideker T Cytoscape: a software environment for integrated models of biomolecular interaction networks Genome Res 2003 13 11 2498 2504 10.1101/gr.1239303 14597658 PMC403769

