# Supplementary material for: Understanding and predicting synthetic lethal genetic interactions in Saccharomyces cerevisiae using domain genetic interactions
Source: BMC Syst Biol. 2011 May 17;5:73. doi: 10.1186/1752-0509-5-73 (PMC3113237; doi:10.1186/1752-0509-5-73)
Supplement: Additional file 1 — Table S1. Number of predicted SLGIs by MLE approach using different posterior probability threshold [file 1752-0509-5-73-S1.DOC]

**Table S1.** Number of predicted SLGIs by MLE approach using different posterior probability threshold

| Threshold | Number of SLGIs |
| --- | --- |
| 0.9 | 82 |
| 0.8 | 383 |
| 0.7 | 792 |
| 0.6 | 1821 |
| 0.5 | 3267 |
| 0.4 | 5143 |
| 0.3 | 7767 |
| 0.2 | 14096 |
| 0.1 | 32881 |
| 0.09 | 35702 |
| 0.08 | 39785 |
| 0.07 | 44989 |
| 0.06 | 51564 |
| 0.05 | 60807 |
| 0.04 | 77050 |
| 0.03 | 94460 |
| 0.02 | 131199 |
| 0.01 | 205758 |
| 0 | 599752 |
