# Supplementary material for: Understanding and predicting synthetic lethal genetic interactions in Saccharomyces cerevisiae using domain genetic interactions
Source: BMC Syst Biol. 2011 May 17;5:73. doi: 10.1186/1752-0509-5-73 (PMC3113237; doi:10.1186/1752-0509-5-73)
Supplement: Additional file 3 — Table S3-5. Summary statistics of distribution of the similarities between the GO biological processes, cellular component and molecular function of genetic interacting proteins [file 1752-0509-5-73-S3.DOC]

**Table S3.** Summary statistics of distribution of the similarities between the GO biological processes of genetic interacting proteins

| Threshold | T-score  (vs. original GI) | P value  (vs. original GI) | T-score  (vs. All pairs) | P value  (vs. All pairs) |
| --- | --- | --- | --- | --- |
| 0.95 | 2.8616 | 8.18E-03 | 7.0986 | 1.54E-07 |
| 0.9 | 4.1388 | 1.09E-04 | 9.6944 | 6.83E-14 |
| 0.85 | 4.5079 | 1.58E-05 | 11.5580 | 7.19E-21 |
| 0.8 | 3.5569 | 4.27E-04 | 15.3384 | 1.62E-40 |
| 0.75 | 5.1504 | 3.51E-07 | 20.2872 | 6.66E-69 |
| 0.7 | 4.6786 | 3.36E-06 | 22.3357 | 5.43E-85 |
| 0.65 | 6.2352 | 5.66E-10 | 29.9002 | 4.46E-152 |
| 0.6 | 5.4971 | 4.28E-08 | 31.8686 | 9.99E-177 |
| 0.55 | 5.0716 | 4.21E-07 | 33.2063 | 1.48E-193 |
| 0.5 | 2.8398 | 4.53E-03 | 39.0308 | 5.67E-272 |
| 0.45 | 1.8511 | 0.064 | 40.8923 | 1.03E-300 |
| 0.4 | 0.5845 | 0.559 | 46.6709 | 0.00 |
| 0.35 | -0.6147 | 0.539 | 50.3936 | 0.00 |
| 0.3 | -3.5334 | 4.11E-04 | 53.5202 | 0.00 |
| 0.25 | -6.3149 | 2.76E-10 | 60.1044 | 0.00 |
| 0.2 | -11.6473 | 2.92E-31 | 63.5508 | 0.00 |
| 0.15 | -16.2162 | 8.97E-59 | 70.9904 | 0.00 |
| 0.1 | -23.2261 | 1.81E-117 | 82.4446 | 0.00 |
| 0.05 | -31.1076 | 2.91E-205 | 99.6669 | 0.00 |
| 0 | -49.0786 | 0.00 | 185.9402 | 0.00 |

Note: the Resnik method is used to calculate the similarity between the biological process terms

**Table S4.** Summary statistics of distribution of the similarities between the GO cellular component of genetic interacting proteins

| Threshold | T-score  (vs. original GI) | P value  (vs. original GI) | T-score  (vs. All pairs) | P value  (vs. All pairs) |
| --- | --- | --- | --- | --- |
| 0.95 | 2.4239 | 0.0226 | 5.4338 | 1.07E-05 |
| 0.9 | 4.0408 | 1.53E-04 | 7.6053 | 2.31E-10 |
| 0.85 | 4.9111 | 3.03E-06 | 9.8846 | 5.60E-17 |
| 0.8 | 4.8803 | 1.62E-06 | 13.7995 | 1.64E-34 |
| 0.75 | 5.5758 | 3.74E-08 | 17.6206 | 1.89E-55 |
| 0.7 | 5.6365 | 2.39E-08 | 20.1136 | 5.25E-72 |
| 0.65 | 6.9807 | 4.23E-12 | 26.7665 | 3.22E-127 |
| 0.6 | 5.4308 | 6.21E-08 | 27.8059 | 2.90E-141 |
| 0.55 | 4.8200 | 1.52E-06 | 29.0693 | 2.93E-155 |
| 0.5 | 2.8470 | 4.43E-03 | 33.9117 | 1.57E-214 |
| 0.45 | 2.0025 | 0.0453 | 35.5617 | 3.54E-237 |
| 0.4 | 0.2845 | 0.7760 | 39.9349 | 3.08E-301 |
| 0.35 | -1.7901 | 0.0735 | 41.7910 | 0.00 |
| 0.3 | -4.7863 | 1.71E-06 | 44.2991 | 0.00 |
| 0.25 | -8.2880 | 1.22E-16 | 48.8099 | 0.00 |
| 0.2 | -12.9517 | 3.19E-38 | 53.1532 | 0.00 |
| 0.15 | -16.7738 | 1.04E-62 | 59.9635 | 0.00 |
| 0.1 | -24.262 | 1.07E-127 | 67.8925 | 0.00 |
| 0.05 | -32.1225 | 6.40E-218 | 77.9517 | 0.00 |
| 0 | -55.8164 | 0.00 | 43.4725 | 0.00 |

Note: the Resnik method is used to calculate the similarity between the cellular component terms

**Table S5.** Summary statistics of distribution of the similarities between the GO molecular function of genetic interacting proteins

| Threshold | T-score  (vs. original GI) | P value  (vs. original GI) | T-score  (vs. All pairs) | P value  (vs. All pairs) |
| --- | --- | --- | --- | --- |
| 0.95 | 2.4895 | 0.0204 | 3.7151 | 1.14E-03 |
| 0.9 | 4.1629 | 1.15E-04 | 5.8254 | 3.43E-07 |
| 0.85 | 2.7182 | 7.78E-03 | 5.2245 | 1.03E-06 |
| 0.8 | 3.3616 | 8.92E-04 | 7.5871 | 6.66E-13 |
| 0.75 | 3.9179 | 1.03E-04 | 9.3705 | 4.77E-19 |
| 0.7 | 4.6401 | 4.27E-06 | 10.8787 | 4.29E-25 |
| 0.65 | 7.5061 | 1.25E-13 | 15.3320 | 1.44E-47 |
| 0.6 | 7.7377 | 1.82E-14 | 16.9870 | 2.03E-58 |
| 0.55 | 7.1848 | 9.68E-13 | 17.4099 | 7.03E-62 |
| 0.5 | 5.4622 | 5.05E-08 | 18.8874 | 5.92E-74 |
| 0.45 | 4.7483 | 2.12E-06 | 19.5387 | 1.88E-79 |
| 0.4 | 2.9593 | 0.0031 | 21.2428 | 1.80E-94 |
| 0.35 | 3.0087 | 0.0026 | 23.4789 | 4.78E-115 |
| 0.3 | 2.1694 | 0.0301 | 25.6045 | 6.41E-137 |
| 0.25 | -0.0979 | 0.9220 | 27.3813 | 1.31E-157 |
| 0.2 | -2.4949 | 0.0126 | 28.9940 | 7.19E-178 |
| 0.15 | -6.1706 | 6.99E-10 | 30.3384 | 3.11E-196 |
| 0.1 | -9.5693 | 1.30E-21 | 35.3075 | 1.82E-266 |
| 0.05 | -12.8642 | 1.49E-37 | 42.3348 | 0.00 |
| 0 | -17.3589 | 2.94E-66 | 110.0738 | 0.00 |

Note: the Resnik method is used to calculate the similarity between the molecular function terms
